# Supplementary material for: Influence of Bone Substitutes on Mesenchymal Stromal Cells in an Inflammatory Microenvironment
Source: Int J Mol Sci. 2022 Dec 27;24(1):438. doi: 10.3390/ijms24010438 (PMC9820717; doi:10.3390/ijms24010438)
Supplement: Supplementary file 1 [file ijms-24-00438-s001.zip › ijms-2075657-supplementary.pdf]

Supplementary data

Supplementary Figure S1

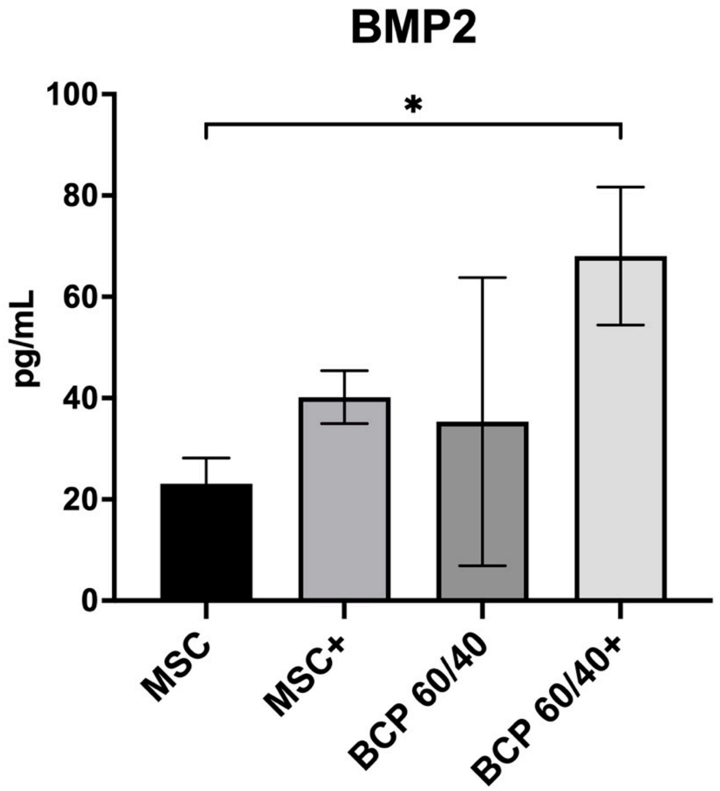

Figure S1: ELISA for BMP2 protein. Concentration of BMP2 protein in different culture conditions after 72 h; + indicates cytokine stimulation. Data represent mean ± SD; * $p < 0.05$.

Supplementary Figure S2

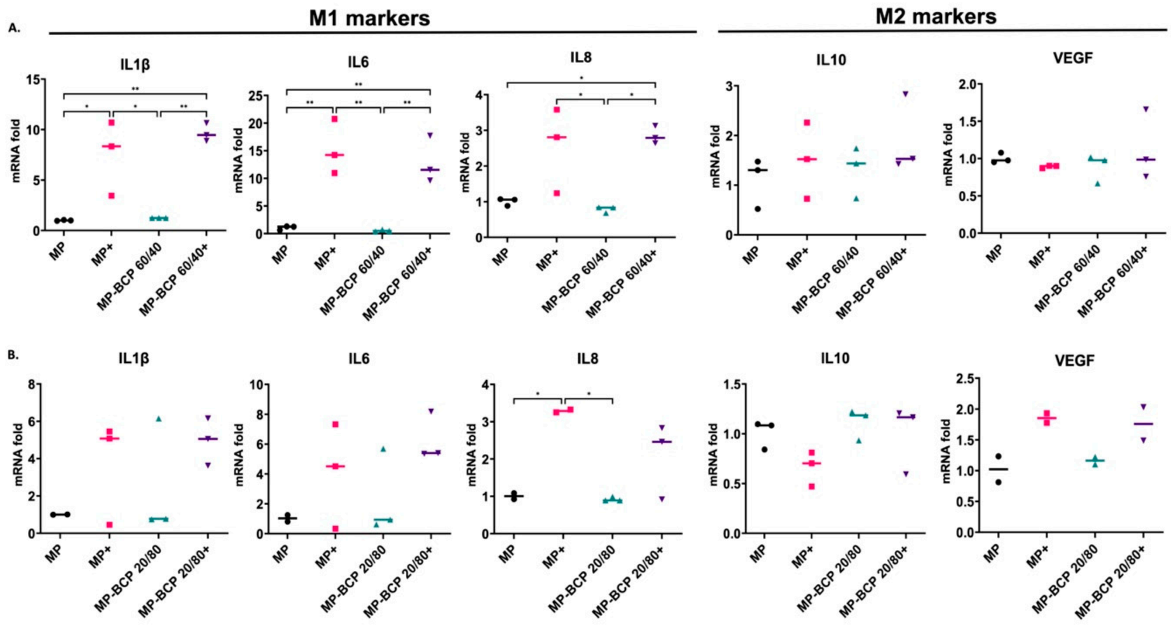

Figure S2: Relative mRNA expression (fold changes) of M1 and M2 related genes in MP cultured with MSC seeded on BCP 60/40 (A) or BCP 20/80 (B) (n = 3). MP, monolayer MSC cocultured with MP; + represents cytokine stimulation. Statistical analyses are based on one-way ANOVA with Tukey's multi comparison tests on delta-Ct values; * $p < 0.05$; ** $p < 0.001$; *** $p = 0.0001$; **** $p < 0.0001$.

Supplementary Table S1: Real time PCR primers used in the study

Gene	TaqMan® Assay ID	Amplicon length
**Reference gene**		
GAPDH	Hs 02758991_g1	93
**Osteogenesis**		
RUNX2	Hs01047973_m1	86
BMP2	Hs00154192_m1	60
**Healing**		
VEGF	Hs00900055_m1	59
IL10	Hs00961622_m1	74
**Inflammation**		
IL6	Hs00174131_m1	95
IL8	Hs00174103_m1	101
**Remodeling**		
RANKL (TNFSF11)	Hs00243522_m1	67
OPG (TNFRSF11B)	Hs00900358_m1	74

GAPDH glyceraldehyde 3-phosphate dehydrogenase, RUNX2 runt-related transcription factor 2, BMP2 bone morphogenetic protein 2, VEGF, vascular endothelial growth factor, IL6 interleukin 6, IL8 interleukin 8, IL10 interleukin 10, RANKL Receptor activator of nuclear factor kappa-B ligand, OPG osteoprotegerin.

Supplementary Table S2: Multiplex cytokine assay panel

Abbreviation	Cytokine
bFGF/FGF2	Basic fibroblast growth factor
GCSF	Granulocyte colony stimulating factor
GMCSF	Granulocyte-macrophage colony-stimulating factor
Eotaxin/CXCL1	Eotaxin-1
IL1β	Interleukin 1 beta
IL1ra	Interleukin 1 receptor a
IL2	Interleukin 2
IL4	Interleukin 4
IL5	Interleukin 5
IL6	Interleukin 6
IL7	Interleukin 7
IL8	Interleukin 8
IL9	Interleukin 9
IL10	Interleukin 10
IL12	Interleukin 12
IL13	Interleukin 13
IL15	Interleukin 15

IL17	Interleukin 17
PDGFBB	Platelet-derived growth factor BB
RANTES/CCL5	Regulated on activation, normal T cell expressed and secreted
IP10/CXCL10	C-X-C motif chemokine ligand 10
VEGF	Vascular endothelial growth factor
MCP1/CCL2	Monocyte chemoattractant protein-1
MIP1α/CCL3	Macrophage Inflammatory Protein-1 alpha
MIP1β/CCL4	Macrophage Inflammatory Protein-1 beta
TNFα	Tumor necrosis factor alpha
INFγ	Interferon gamma
